# Pressure induced superconductivity in the antiferromagnetic Dirac material BaMnBi_2_

**DOI:** 10.1038/s41598-017-01967-y

**Published:** 2017-05-09

**Authors:** Huimin Chen, Lin Li, Qinqing Zhu, Jinhu Yang, Bin Chen, Qianhui Mao, Jianhua Du, Hangdong Wang, Minghu Fang

**Affiliations:** 1 Hangzhou Key Laboratory of Quantum Matter, Department of Physics, Hangzhou Normal University, Hangzhou, 310036 China; 2 Department of Physics, Zhejiang University, Hangzhou, 310027 China; 3 Collaborative Innovation Center of Advanced Microstructures, Nanjing, 210093 China

## Abstract

The so-called Dirac materials such as graphene and topological insulators are a new class of matter different from conventional metals and (doped) semiconductors. Superconductivity induced by doing or applying pressure in these systems may be unconventional, or host mysterious Majorana fermions. Here, we report a successfully observation of pressure-induced superconductivity in an antiferromagnetic Dirac material BaMnBi_2_ with *T*_*c*_ of ~4 K at 2.6 GPa. Both the higher upper critical field, *μ*_0_ *H*_*c*2_(0) ~ 7 Tesla, and the measured current independent of *T*_*c*_ precludes that superconductivity is ascribed to the Bi impurity. The similarity in *ρ*_*ab*_(*B*) linear behavior at high magnetic fields measured at 2 K both at ambient pressure (non-superconductivity) and 2.6 GPa (superconductivity, but at the normal state), as well as the smooth and similar change of resistivity with pressure measured at 7 K and 300 K in zero field, suggests that there may be no structure transition occurred below 2.6 GPa, and superconductivity observed here may emerge in the same phase with Dirac fermions. Our findings imply that BaMnBi_2_ may provide another platform for studying SC mechanism in the system with Dirac fermions.

## Introduction

The so-called Dirac materials such as graphene and topological insulators (TIs), in which a linear energy dispersion similar to the spectrum of relativistic Dirac particles was found, are a new class of matter different from conventional metals and (doped) semiconductors^1^, in which a quadratic dispersion was commonly observed. According to the Bardeen-Cooper-Schrieffer (BCS) theory in the weak-coupling limit, the low density of states (DOS) at the Fermi level for un-doped or lightly doped Dirac materials is usually harmful to superconductivity (SC) emerging in these materials. However, SC has been observed in many Dirac materials, such as the Cu-doped TI Bi_2_Se_3_^2–4^, Sn_1−*x*_In_*x*_Te^5^, as well as the pressurized TIs Bi_2_Se_3_^6^, Bi_2_Te_3_^7^ and Sb_2_Te_3_^8, 9^. In the Cu_*x*_Bi_2_Se_3_ superconductor (its superconducting transition temperature, *T*_*c*_ ~ 3.4 K), the presence of a zero-bias conductance peak (ZBCP) in the point-contact spectra on its cleaved surface may give evidence for a topological SC^4^. The very recent results of the nuclear magnetic resonance (NMR) measurements^10^ for Cu_*x*_Bi_2_Se_3_ established a spin-triplet SC in this compound. Especially, the topological superconductors are expected to host unusual Majorana fermions on its surface^11–13^, which are peculiar in that the particles are their own antiparticles, and are originally conceived as mysterious neutrinos^14^. The realization of Majorana fermions in condensed matter is of significant interest because of their novelty as well as the potential for quantum computing^14^. Triggered by these researches, now searching for SC and exploring the nature of SC in various Dirac materials become a very hot field in the condensed matter physics.

**Figure 1:**
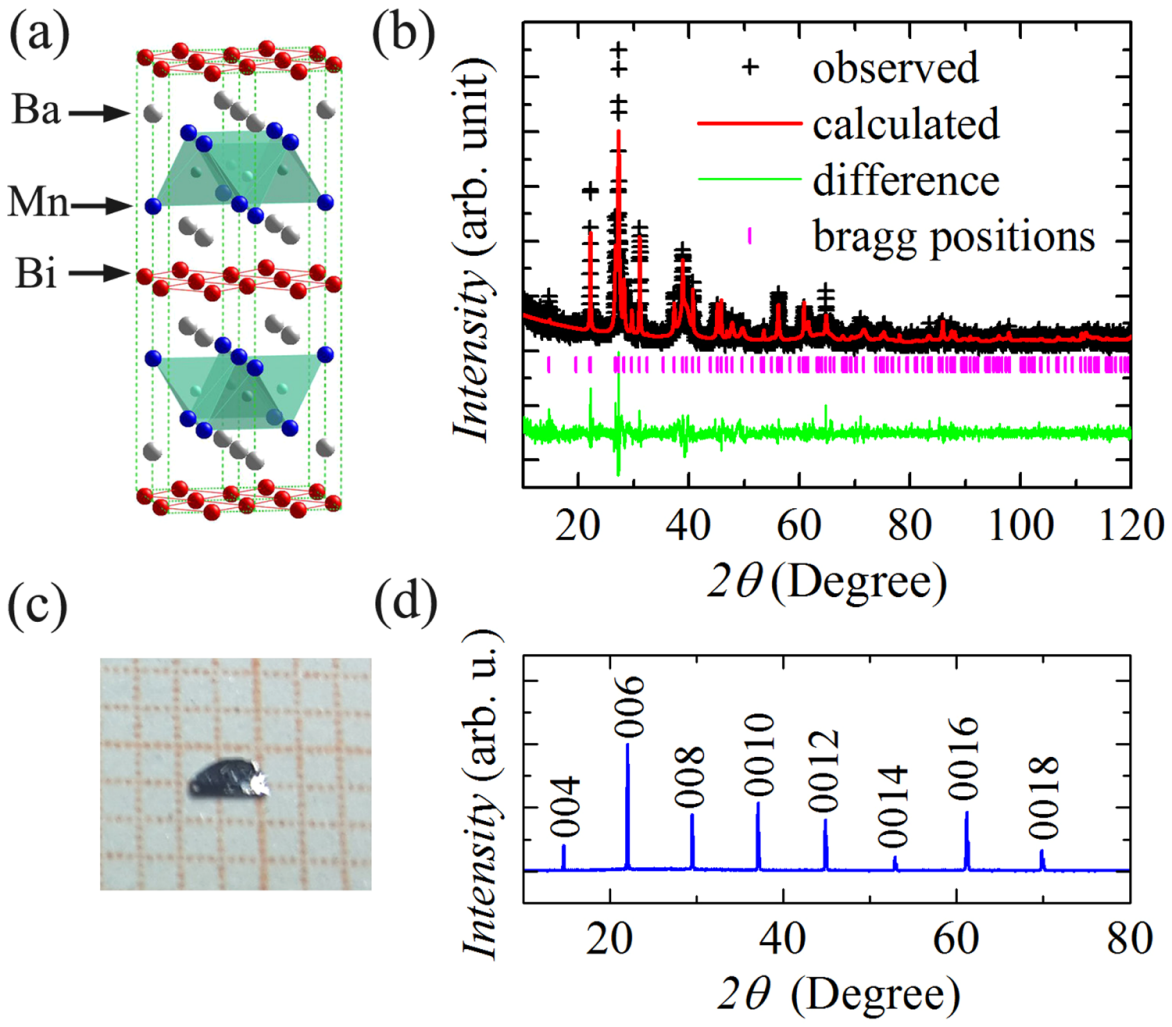
(**a**) Crystal structure of BaMnBi_2_. (**b**) XRD pattern of powder obtained by grinding BaMnBi_2_ crystals. Its Rietveld refinement is shown by the solid lines. (**c**) A photo of BaMnBi_2_ crystal. (**d**) Single-crystal XRD pattern of BaMnBi_2_.

BaMnBi_2_^15^ consists of a MnBi layer with edge-sharing MnBi_4_ tetrahedrons and a two-dimensional (2D) Bi square net stacked with Ba atoms as shown in Fig. 1(a). As discussed by Park *et al*.^16^ for the isostructural SrMnBi_2_, the ionic Ba layers electronically separate the MnBi layers and the Bi square net because of the low electronegativity of Ba. In the [MnBi]^−^ layer, Mn^2+^ has a half filled 3*d* ^5^ configuration. The strong Hund coupling of Mn^2+^ leads to an antiferromagnetic ground state in MnBi layers^17^. Based on the first-principles calculations, angle-resolved photoelectron spectroscopy (ARPES) measurements^15, 16, 18–23^, it has been confirmed that the Mn bands are placed away from the Fermi level (*E*_*F*_), the states near *E*_*F*_ are dominated by the Bi states in the square net where the Dirac-like energy dispersion is seen at *k*_0_ = (0.208, 0.208). There are two identical Bi atoms per unit cell because Ba atoms below and above the square net result in unit cell doubling. This leads to folding of the dispersion Bi 6*p* bands and makes the two Bi *p*_*x*,*y*_ bands cross each other. The Ba-Bi hybridization lifts the degeneracy of the folded bands except the momentum space along the Γ-*M* symmetry line, resulting in the formation of the Dirac cone. A small gap near the Dirac point appears due to the presence of the spin-orbital coupling (SOC). The magnetotransport properties, nonzero Berry phase, small cyclotron mass measurements^15^ on BaMnBi_2_ also confirm the presence of Dirac fermions in Bi square net. The quantum oscillations^15^ in the Hall channel suggest the presence of both electron and hole pockets, whereas Dirac and parabolic states coexist at the *E*_*F*_. On the other hand, the Dirac materials *An*MnBi_2_ (*An* = Ca, Sr, Ba) are suggested to be promising parent compounds for superconductivity, due to their striking similarity to the superconducting iron pnictides. Up to now, superconductivity has not been observed in these materials, although several authors suggested that chemical doping may introduce superconductivity in these systems^16, 20^. Here, we report a successfully observation of pressure-induced superconductivity in an antiferromagnetic Dirac material BaMnBi_2_ with *T*_*c*_ of ~4 K, at 2.6 GPa. Our findings imply that BaMnBi_2_ may provide another platform for studying SC mechanism in the system with Dirac fermions.

## Results and Discussion

We grew the BaMnBi_2_ crystals using a self-flux method (see the method in details). The x-ray diffraction (XRD) pattern [see Fig. 1(b)] of BaMnBi_2_ powder created by grinding pieces of crystals confirms its tetrahedral structure with space group *I*4/*mmm*, and its Rietveld refinement [see Fig. 1(b)] gives the lattice parameters of *a* = 4.628(1) *Å* and *c* = 24.092(4) *Å*, in consistent with the previous results reported by Li *et al*.^15^. Single crystal XRD pattern [see Fig. 1(d)] shows that the basal plane of a cleaved crystal is the crystallographic *ab*-plane. The energy-dispersive x-ray spectroscopy (EDX) results indicate that the crystals are rather homogenous and the determined average atomic ratios are Ba:Mn:Bi = 1.02:0.99:2.00 when fixing Bi stoichiometry to be 2, confirming the stoichiometry of BaMnBi_2_.

**Figure 2:**
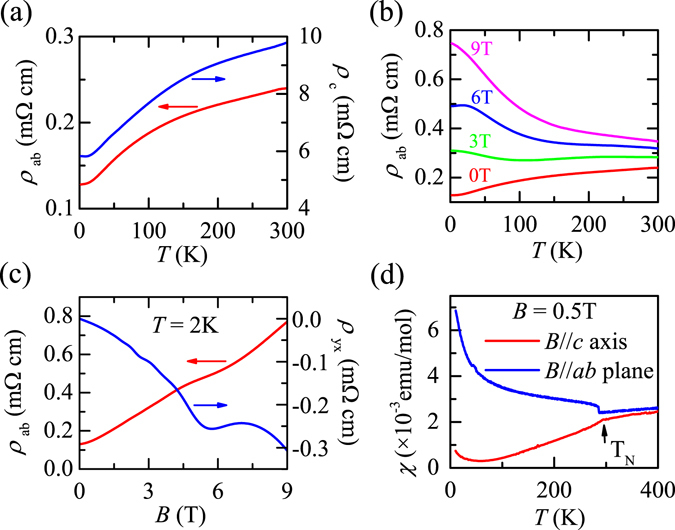
(**a**) Temperature dependence of the in-plane (*ρ*_*ab*_) and out-plane (*ρ*_*c*_) resistivity. (**b**) Temperature dependence of the *ρ*_*ab*_ under various fields. (**c**) *ρ*_*ab*_ and Hall resistivity (*ρ*_*yx*_) as a function of the out-of plane magnetic field at *T* = 2 K. (**d**) Temperature dependence of the magnetic susceptibility with field parallel and perpendicular to *c* axis for BaMnBi_2_ crystal.

The physical properties of BaMnBi_2_ crystal are summarized in Fig. 2. The temperature dependence of the in-plane, *ρ*_*ab*_, and out-plane, *ρ*_*c*_, resistivity at ambient pressure is shown in Fig. 2(a). Both *ρ*_*ab*_(*T*) and *ρ*_*c*_(*T*) exhibit a metallic behavior. However, *ρ*_*c*_ is almost two orders of magnitude larger than *ρ*_*ab*_, *i*.*e*. at 300 K *ρ*_*c*_/*ρ*_*ab*_ ≈ 41. The strong anisotropy in resistivity is consistent with its quasi-2D electronic structure in BaMnBi_2_ as discussed above. Figure 2(b) shows *ρ*_*ab*_(*T*) curves at various magnetic fields. It is clear that the magnetic field induced metal-insulator transition occurs, such as, at 6 Tesla, its *ρ*_*ab*_ increases with decreasing temperature, and reaches a constant at the low temperatures, *i*.*e*. exhibiting a semiconductor-like behavior. Even at room temperature (300 K), there is also a large different in resistance at different magnetic fields, indicating that BaMnBi_2_ exhibits a large magnetoresistance. Figure 2(c) shows the *ρ*_*ab*_ and Hall resistivity, *ρ*_*yx*_, as a function of the out-of plane magnetic field at *T* = 2 K. Clear Shubnikov-deHaas (SdH) oscillations in both the *ρ*_*ab*_(*B*) and Hall resistivity *ρ*_*yx*_(*B*) are observed, indicating the presence of small fermi surface in BaMnBi_2_. Figure 2(d) displays the temperature dependence of magnetic susceptibility, *χ*(*T*). At the Néel temperature, *T*_*N*_ = 288 K, an antiferromagnetic (AFM) transition was clearly observed. All these results are quite similar with that reported by Li *et al*.^15^, also suggesting the existence of the Dirac fermions in our crystals. However, compared with their results, a major difference is found in Hall resistivity, which is negative, indicating that the electrons are dominant carriers in our crystals, which may origin from the slight shift of the *E*_*F*_ near the Dirac cone by a light electron-doping.

**Figure 3:**
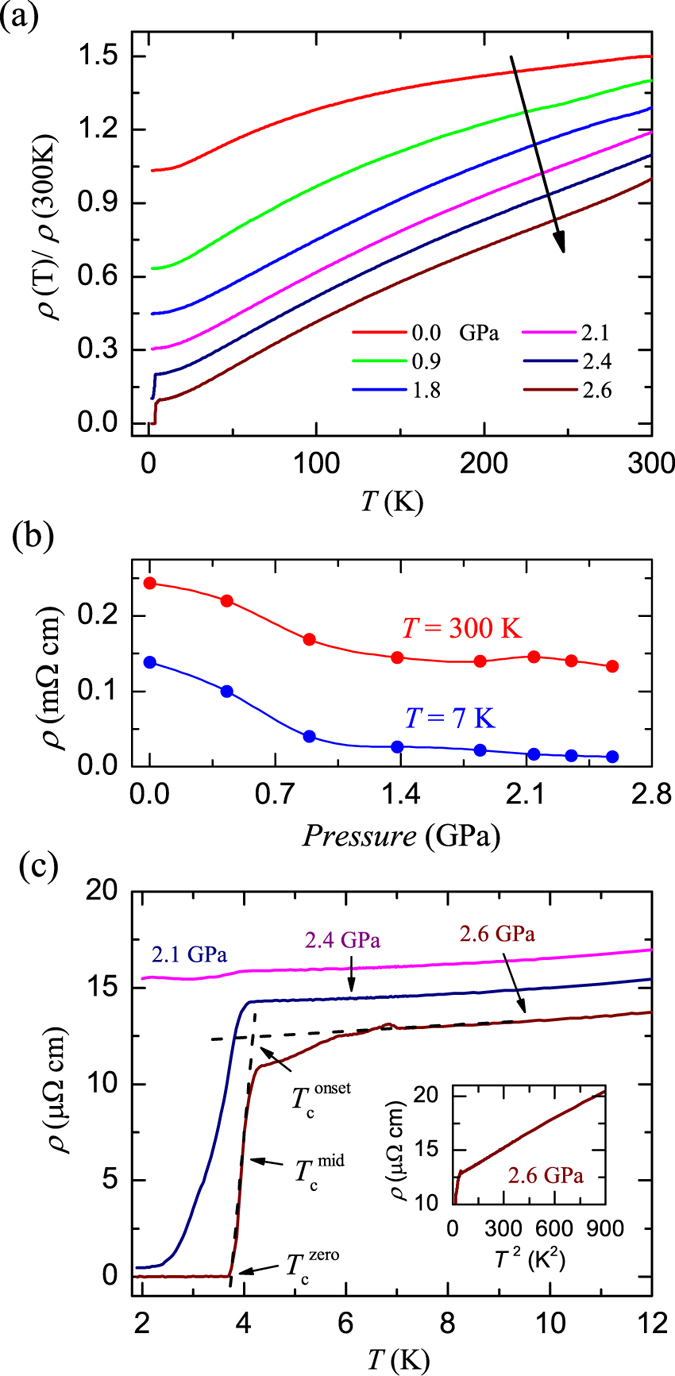
(**a**) Temperature dependence of the normalized in-plane resistivity, *ρ*(*T*)/*ρ*(300 *K*), for a BaMnBi_2_ crystal under various pressures up to 2.6 GPa. Note that each subsequent data set is shifted upward by 0.1 for clarity. (**b**) Pressure dependence of *ρ* measured at 300 K and 7 K. (**c**) The low temperature *ρ*(*T*) curves measured at various pressures. The criteria used to determine the onset, middle and zero temperatures for the superconducting transitions. Inset: *ρ* as a function of *T* ^2^ below 30 K under 2.6 GPa.

The key result of this work is shown in Fig. 3. The evolution of the normalized resistivity, *ρ*_*ab*_(T)/*ρ*_*ab*_ (300 K), as a function of temperature at various pressures for BaMnBi_2_ crystal is shown in Fig. 3(a). It can be seen that the metallic behavior in *ρ*_*ab*_(*T*) at higher temperatures, *i*.*e*., *ρ*_*ab*_ monotonously deceases with deceasing temperature, is robust to pressure. At *T*_*N*_, no anomaly in *ρ*_*ab*_(*T*) due to AFM transition from the Mn^2+^ moments was observed under all the applied pressures, which is in consistent with the conduction in BaMnBi_2_ determined by the Bi states in the square net as discussed above, therefore makes it impossible to figure out the pressure dependence of *T*_*N*_ by using only the resistance measurements. It is very interesting that a clear superconducting transition was observed at pressures above 2.4 GPa, even at 2.1 GPa a slight drop in *ρ*_*ab*_ can be distinguished. The definition of superconducting transition temperatures of onset, midpoint, and zero resistance, for 2.6 GPa data are shown in Fig. 3(c), and ${T}_{c}^{onset}$ = 4.13 K, ${T}_{c}^{mid}$ = 3.97 K and ${T}_{c}^{zero}$ = 3.69 K were obtained. Compared with the data of 2.4 GPa, it should be pointed out that there are other two kinks in resistance for 2.6 GPa at *T* = 5.86 K and 6.81 K, respectively. The origin of these two kinks is not clear yet, however, we suggest that they may be associated with other two superconducting transitions, since they are smoothed out by the applied field. This result indicates that SC with higher *T*_*c*_ may emerges at higher pressures. At the same time, we plot the resistivity data at 2.6 GPa as a function of *T* ^2^ up to 30 K, as shown in the inset of Fig. 3(c), in which the good *ρ*_*ab*_(*T*) ~ *T* ^2^ behavior above *T*_*c*_ indicates its fermi liquid ground state. In order to check whether the structure transition occurs by applying pressure, we plot the resistivity data at 300 K and 7 K as a function of pressure, as shown in Fig. 3(b). It can be seen that the resistivity deceases smoothly with increasing pressure. The smooth and similarity of *ρ*_*ab*_ changing with pressure at both temperatures may preclude the possibility of structure transition occurring below 2.6 GPa. However, further work is still needed to confirm this result.

**Figure 4:**
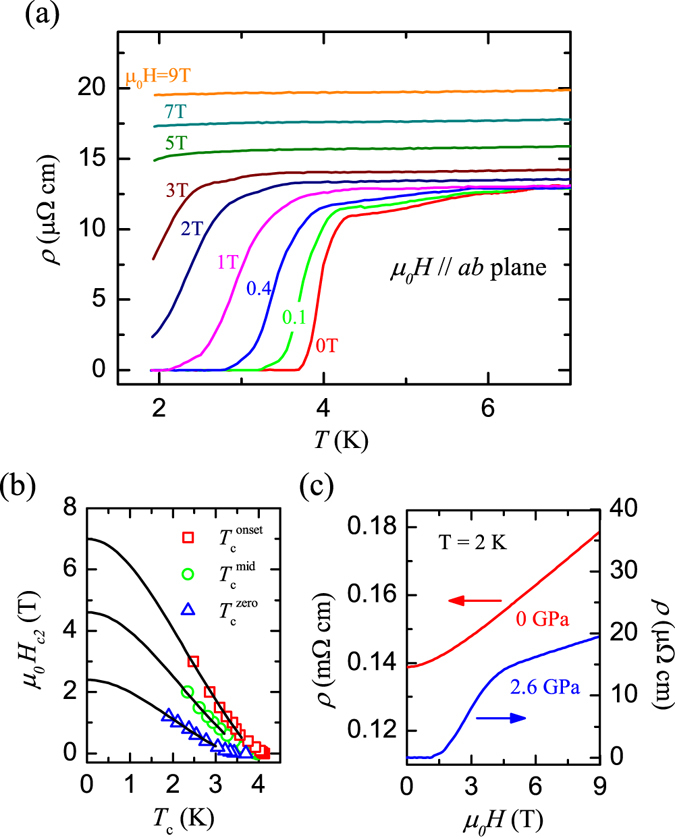
(**a**) Temperature dependence of the *ab*-plane resistivity measured at 2.6 GPa in magnetic fields up to 9 T for BaMnBi_2_ crystal. (**b**) Upper critical field *μ*_0_ *H*_*c*2_ *vs T*_*c*_ determined by using the superconducting transition temperatures in (**a**). (**c**) Magnetic field dependence of *ρ* measured at 2 K under ambient pressure and 2.6 GPa. The field is parallel to *ab*-plane.

About the origin of the superconductivity, we noted that the *T*_*c*_ for BaMnBi_2_ is very close to that of Bi single crystal under high pressure^24, 25^. To assure what has been observed in Fig. 3 is indeed a superconducting transition and to exclude the SC originating from Bi impurity, we further conducted the measurements at variant external magnetic field at 2.6 GPa. As shown in Fig. 4(a), with the increase of magnetic field, the superconducting transition temperature decreases, and the width of superconducting transition increases gradually from 0.4 K at zero field to 1.2 K at 1 T. The upper critical field *μ*_0_ *H*_*c*2_ as a function of ${T}_{c}^{onset}$, ${T}_{c}^{mid}$, and ${T}_{c}^{zero}$ is plotted in Fig. 4(b), respectively. It can be seen that *μ*_0_ *H*_*c*2_(*T*) near *T*_*c*_ has a positive curvature, a characteristic of two band clean-limit type-II superconductors, like YNi_2_B_2_C, LuNi_2_B_2_C^26^, MgB_2_^27^, or TlNi_2_Se_2_^28^. According to the Ginzburg-Landau theory, the zero temperature upper critical field *H*_*c*2_(0) can be estimated by using the formula *H*_*c*2_(*T*) = *H*_*c*2_(0)(1 − *t* ^2^)/(1 + *t* ^2^), where *t* is the reduced temperature *t* = *T*/*T*_*c*_. Using the ${T}_{c}^{onset}$, ${T}_{c}^{mid}$, and ${T}_{c}^{zero}$, the fitting result yields the value of *μ*_0_ *H*_*c*2_(0) = 7.0, 4.6, and 2.4 Tesla, respectively. Compared with the critical field of Bi element under high pressure, these values are two orders of magnitude larger. Besides, we also carried out resistivity measurements with different applied currents, and no obvious difference in *T*_*c*_ was observed. So, we conclude that the observed SC is intrinsic to BaMnBi_2_, and can’t be ascribed to the Bi impurity.

In addition to the SC in BaMnBi_2_ at high pressures, the large magnetoresistivity effect [see Fig. 4(a)] due to the presence of Dirac fermions is also survived. Figure 4(c) displays the *ρ*_*ab*_ at 2 K measured at both ambient and 2.6 GPa pressure, respectively, as a function of magnetic field, *B*. It can be seen that the *ρ*_*ab*_ measured at ambient pressure increases with increasing magnetic field, and exhibits a linear behavior at higher field, while *ρ*_*ab*_(*B*) measured at 2.6 GPa has a similar behavior as *B* > 4 Tesla (at the normal state). The similarity in *ρ*_*ab*_(*B*) behavior measured both at ambient pressure and 2.6 GPa in the high magnetic field range indicates that SC observed here emerges in the same phase with Dirac fermions. Therefore, we suspect that the Dirac fermions may preserve under high pressure and play an important role in the SC in BaMnBi_2_. Our findings imply that BaMnBi_2_ may provide another platform for studying SC mechanism in the system with Dirac fermions.

Finally, we should point out that BaMnBi_2_, as an AFM compound of Mn^2+^ 3*d* ^5^ half-filled electrons with *T*_*N*_ = 288 K at ambient pressure, is expected to be a promising parent for SC. As we know, in the cuprates, Fe-pnictides, and heavy-fermion compounds, doping or applying a pressure can suppress the AFM order, then unconventional SC emerges. Here, although we can’t figure out the pressure dependence of *T*_*N*_ by using only resistance measurements as discussed above, we suspect that the AFM order in BaMnBi_2_ seems impossible to be suppressed by such low pressure (≤2.6 GPa), just like the robust antiferromagnetism under high pressure in (Ba_0.61_K_0.39_)Mn_2_Bi_2_^29^, which contains the similar Mn_2_Bi_2_ layers. Taken this assumption, then the SC observed here should originate from the electrons in Bi square net, which host both Dirac and parabolic states. Therefore, SC in the pressurized BaMnBi_2_ may coexist with the AFM order. It is urgently needed to figure out the relationship among SC, AFM and Dirac fermions, as well as the symmetry of Cooper pairs in this system in the near future.

In summary, we present resistivity measurements on the antiferromagnetic (*T*_*N*_ = 288 K) Dirac material BaMnBi_2_ under various pressures up to 2.6 GPa. At *T*_*N*_, no anomaly in *ρ*_*ab*_(*T*) due to AFM transition was observed, which makes it impossible to figure out the pressure dependence of *T*_*N*_ by using only the resistance measurements. *ρ*_*ab*_(*T*) shows a clear superconducting transition with *T*_*c*_ ~ 4 K at 2.4 GPa, but does not drop to zero. Under 2.6 GPa, a sharp superconducting transition with ${T}_{c}^{onset}$ = 4.13 K, ${T}_{c}^{mid}$ = 3.97 K and ${T}_{c}^{zero}$ = 3.69 K was observed. Both the higher upper critical field, *μ*_0_ *H*_*c*2_(0) ~ 7 Tesla, close to its Pauli limit *H*_*p*_ = 1.84 *T*_*c*_ ≈ 7.2 T, and the measured current independent of *T*_*c*_ precludes that SC is ascribed to the Bi impurity. The similarity in *ρ*_*ab*_(*B*) linear behavior at high magnetic fields measured at 2 K both at ambient pressure and 2.6 GPa indicates that SC observed here would emerge in the same phase with Dirac fermions. Our findings imply that BaMnBi_2_ may provide another platform for studying SC mechanism in the system with Dirac fermions.

## Methods

High quality BaMnBi_2_ single crystals were grown using self-flux method. First, Ba chunks, and Mn, Bi powders were mixed according to an appropriate stoichiometry and were put into alumina crucibles and sealed in an evacuated silica tube. The mixture was heated up to 850 °C and kept for 3 hours. Then the melting mixture was cooled down to 450 °C with a rate of 3 °C/h. Finally the furnace was cooled to room temperature after shutting down the power. The structure of single crystals was characterized by powder X-ray diffraction (XRD) measurement at ambient pressure. To avoid exposure to air, the sample was sealed using *N*-grease during the XRD data collecting. The elemental analysis was performed using an energy-dispersive x-ray spectroscopy (EDX) in a Zeiss Supra 55 scanning electron microscope. The measurements of resistivity under various hydrostatic pressures below 3 GPa, were carried out in the *QuantumDesign* Physical Properties Measurement System PPMS-9. The magnetic susceptibility *χ*(T) was measured using the *QuantumDesign* MPMS-SQUID. Pressure was generated in a Teflon cup filled with Fluorinert FC-75, which was inserted into a nonmagnetic, piston-cylinder type, Be-Cu pressure cell with a core made of NiCrAl alloy. The pressure was determined at low temperature by monitoring the shift in the *T*_*c*_ of pure lead.
